# Investigating the Influence of Limited CT Scan Coverage Using an Automated Workflow with Biofidelic Sideways Fall FE Models

**DOI:** 10.1007/s10439-025-03938-1

**Published:** 2026-01-13

**Authors:** Alexander Baker, Ingmar Fleps, Fang-Chi Hsu, Pierre Guy, Peter Cripton, Stephen J. Ferguson, Benedikt Helgason

**Affiliations:** 1 Laboratory for Orthopaedic Technology, Department of Health Sciences and Technology, Institute for Biomechanics, ETH Zürich, Zürich, Switzerland; 2 Biostatistics and Data Science, Wake Forest University School of Medicine, Winston-Salem, North Carolina USA; 3 Centre for Aging SMART, University of British Columbia, Vancouver, British Columbia Canada; 4 Division of Orthopaedic Trauma, Department of Orthopaedics, University of British Columbia, Vancouver, British Columbia Canada; 5 Orthopaedic and Injury Biomechanics Group, School of Biomedical Engineering and Departments of Mechanical Engineering and Orthopaedics, University of British Columbia, Vancouver, British Columbia Canada

**Keywords:** Osteoporosis, Fragility fracture, Femur strength, Computed tomography, Finite element modeling, Sideways fall, Automation

## Abstract

Sophisticated biofidelic finite element (FE) models of sideways falls are an emerging tool for predicting hip fracture risk. We adapted an existing experimental setup for in-silico trials by creating an automated workflow to build FE models of the experiment and characterizing it with respect to the effects of limited CT scan coverage. Limited CT scan coverage was simulated by shortening the femur (25–175mm distal to the greater trochanter), misaligning the femur up to 5 degrees, and using a morphed template pelvis. We compared impact force error (*E*_*BL*_) of the FE results to the existing experimental results. We then characterized the limited CT scan coverage with respect to the impact force (*E*_*I*_), femur force (*E*_*F*_), and fragility ratios based on the impact force (*E*_*FRI*_) and the femur force (*E*_*FRF*_) by comparing results to a model without any of these scan-related errors introduced. In general, the baseline simulations agreed well with the experiments (*E*_*BL*_*: μ* = − 0.007 kN, σ = 0.409 kN). When scan coverage errors were introduced, the errors were small (*E*_*I*_: *μ* = 0.082kN, σ = 0.232kN; *E*_*F*_: μ = 0.063 kN, σ = 0.228 kN; *E*_*FRI*_: *μ* = − 0.00, σ = 0.058; *E*_*FRF*_: *μ* = − 0.012, σ = 0.074). The pelvis template used explained the most variance of the output measures (*E*_*I*_ *R*^2^ = 0.876; *E*_*F*_ *R*^2^ = 0.880; *E*_*FRI*_ *R*^2^ = 0.901; *E*_*FRF*_*R*^2^ = 0.884). These results indicate that this automated methodology is suitable for typical scan coverages encountered clinically, and future work should use this workflow to explore fragility fractures in larger clinical cohorts.

**Supplementary Information:**

The online version contains supplementary material available at https://doi.org/10.1007/s10439-025-03938-1.

## Introduction

Fragility fractures of the hip are a growing concern for the elderly population. While hip fractures comprise only approximately one-fifth [1] to one-third [2] of all osteoporotic fractures, the outcomes have high socioeconomic impact. At six months post-fracture, the mortality rate is between 11 and 23%; at 12 months post-fracture, the mortality rate is 22–29% [3]. After hospitalization due to a hip fracture, only 70% will be discharged to their original accommodation, and less than 50% will have the same pre-fracture walking ability. [4].

Estimated bone strength via finite element (FE) models are commonly used as a surrogate measure for hip fracture risk [5–11] Compared to dual X-ray absorptiometry measured areal bone mineral density (aBMD), FE models perform equivalently or slightly better than aBMD when discriminating between fracture and non-fracture in clinical cohorts [7, 10, 12–14] More recently, there have been efforts to simulate an entire fall event using FE models [15, 16], and initial efforts have shown this type of modeling to be better at discriminating fracture in likely fallers compared to aBMD [5].

At the same time, increasingly complicated ex-vivo experiments and associated FE simulations [15–18] have shown great promise in predicting fracture during a sideways fall. The experimental setup from Fleps et al. [18] includes the femur, pelvis, acetabular joint capsules, and a surrogate soft tissue, and is perhaps the most realistic controlled sideways fall experimental setup to date. Additionally, experimentally matched FE models showed great predictive ability in predicting experimental fracture and impact loads. While this setup was originally intended to investigate the mechanics of a fracture during a sideways fall, the accuracy of the complementary FE models suggests that such a setup may be useful for fracture prediction in clinical cohorts. However, these FE models require a large amount of CT scan coverage—most notably full coverage of the pelvis along with a substantial portion of the femur. Due to the need to reduce scan times and reduce radiation exposure, this scan coverage is unlikely to be found in clinical cohorts. Additionally, to adapt this for clinical practice and to perform in-silico trials with a meaningful sample size, the model building process must be automated.

The effect of CT scan sizes is not well researched in the context FE models or biomechanical simulations. Instead, there is research into excessive over-scanning which increases patient CT exposure [19]—but yet may be important for FE model creation. There has been substantial research into the effects different CT scanners, CT scanner settings, and in-vivo to in-vitro comparisons [20], but none have focused on the effect of axial scan coverage.

The goal of this study is to investigate the potential drawbacks of adapting the experimental setup introduced by Fleps et al. [18] for large in-silico trials. Specifically, we aimed to (1) introduce and characterize an automated workflow for building sideways fall biofidelic finite element models; (2) evaluate the automated workflow against previously published reference experimental results; (3) investigate what errors are incurred by having limited CT scan coverage—specifically with respect to using a generic pelvis and a femur that is less visible than what is commonly available experimentally; and (4) understand what influences these errors the most.

## Materials and Methods

### Reference Experimental Setup

The reference experiment from Fleps et al. [17] is briefly described here for clarity and context. Six female specimen and five male specimen cadavers were acquired (Table 1). For each specimen, the femurs and pelvis were extracted and placed into a lower-limb fall simulator.

**Table 1:** Specimen information of the reference experiment from Fleps et al. [16], along with measurements of the femoral head radii (left, FHR_L_; right, FHR_R_) and the femoral head to femoral head distance (D_FH_)

Specimen [16]	Sex [16]	Age [16] [Yr]	Weight [16] [kg]	Height [16] [m]	BMI [16] [kg m^−2^]	FHR_L_ [mm]	FHR_R_ [mm]	D_FH_ [mm]
H1389	Female	88	40.8	1.63	15.4	22.8	23.9	174.9
H1390	Female	54	49.0	1.79	15.5	23.3	23.3	182.0
H1391	Female	62	59.0	1.65	21.6	21.7	21.1	160.2
H1392	Female	88	61.3	1.68	21.8	21.9	21.5	157.3
H1394	Female	91	84.0	1.63	31.8	23.6	24.5	177.6
H1395	Female	67	99.8	1.58	40.2	20.9	20.5	176.7
H1397	Male	68	45.4	1.75	14.8	25.2	25.0	166.7
H1399	Male	85	63.5	1.83	19.0	25.0	25.0	162.2
H1401	Male	81	52.2	1.63	19.7	25.7	26.1	172.3
H1402	Male	70	68.1	1.75	22.2	25.9	25.9	174.4
H1406	Male	94	84.0	1.73	28.2	25.5	26.6	187.1

**Fig. 1:**
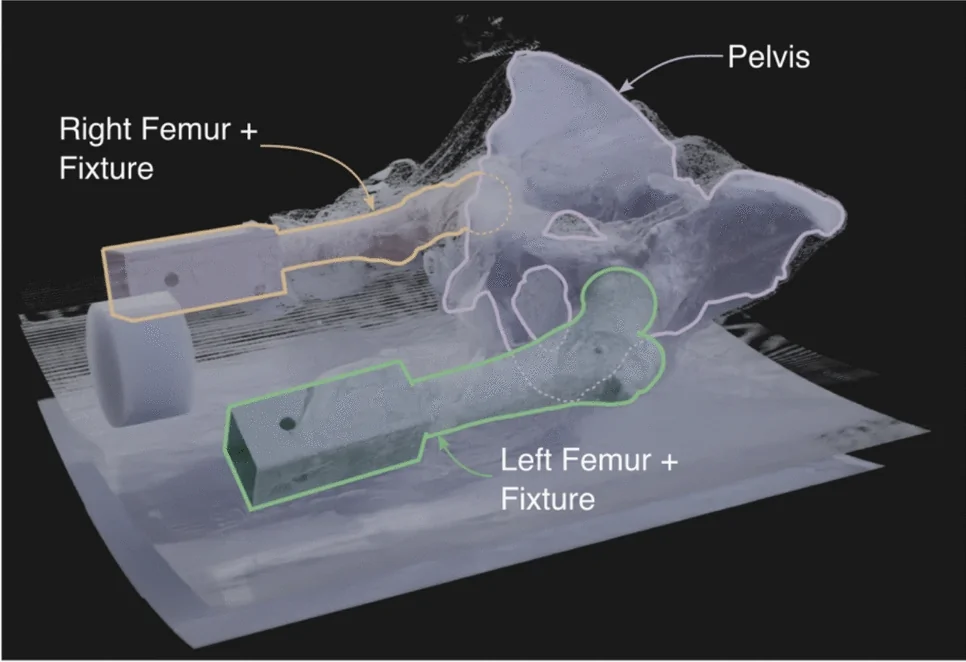
From the CT scan, the femurs, potting fixtures, and pelvis are extracted to build the computational model

Each specimen was cut 175 mm distal to the lateral aspect of the greater trochanter and potted in a PMMA fixture with a target angle of 13° internal rotation. CT Scans were acquired for each specimen after placing the bones in the setup so that an accurate computational model could be built (Fig. 1). The potting fixture determines the frontal plane which, in turn, determines the internal rotation. This also sets the shaft direction as the femur is only extended straight in the experimental setup.

The femur–pelvis construct was embedded in a soft tissue surrogate made of ballistic gel and was subjected to an inverted pendulum impact. The target impact velocity was 3.1 m/s at the greater trochanter.

### Automated Finite Element Model Construction

To investigate the potential effects with a sufficient sample size, a fully automated FE model construction process was implemented. This was based on the simulations previously built by Fleps et al. [16, 17], briefly described here for clarity and context. The pelvis and femurs were modeled using a strain rate-dependent foam model with strain softening to simulate failure [21]. Each element was assigned a material property according to the density derived from the underlying CT scan [21]. Ash density of the bone was assumed to be 60% of the apparent density [22]. Soft tissue properties were modeled using a hyperelastic material model [17]. Models were solved using a commercial, explicit FE solver (LS-Dyna R12, ANSYS).

The automated construction consisted of three main components: (1) morphing a generic pelvis to the correct acetabulum size and femoral head placements; (2) extending the femur to the experimental length; and (3) placing the bones within a representative soft tissue shape and morphing the soft tissue shape into a fall configuration.

### Pelvis Morphing

**Fig. 2:**
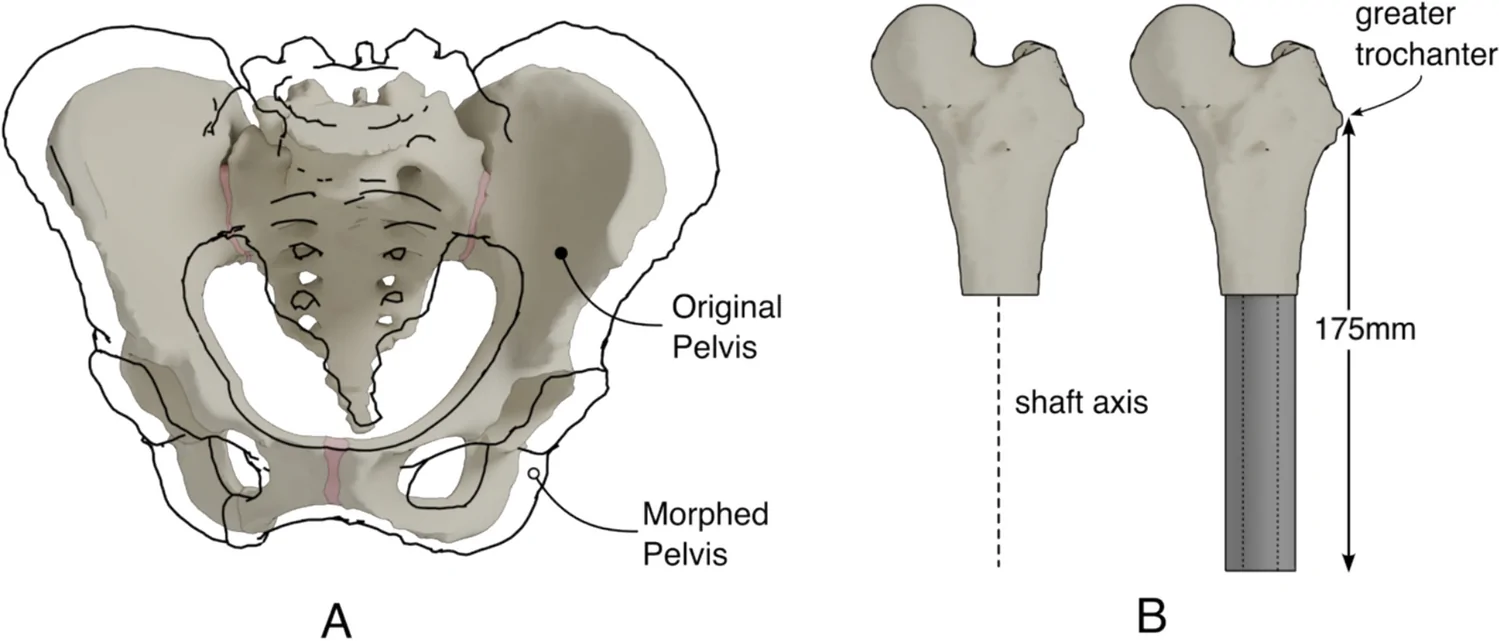
**A** Example of a pelvis morph. Here, the pelvis from H1392 (D_FH_ = 157.3mm) is morphed to the femoral head distance of H1390 (D_FH_ = 182.0mm). The acetabular cups are also morphed to accommodate the different femoral head sizes. **B** Example of the extension of the shaft for femurs when the covered scan is too short

In order to use a pelvis from a different specimen, the pelvis needed to be morphed to accommodate the specimen’s characteristics. This morph was performed in two steps. First, the acetabulum was morphed according to Enns-Bray et al. in order to fit the femoral head of a different specimen, corresponding to FHR_L_ and FHR_R_ (Table 1) [5]. Next, while the acetabulum was rigidly constrained, the pelvis was morphed using an affine-like morph with a commercial preprocessor (ANSA, Beta CAE Systems) such that the acetabular cups from the template pelvis would align with the femoral heads of the target specimen, corresponding to D_FH_ (Table 1). The entire process can be visualized in Fig. 2A.

### Femur Extension

To fit a limited femur within the setup, the femur was extended along the shaft direction, using a hollow cylinder of cortical bone properties. The outside diameter was 26.01mm and the inside diameter was 12.96mm. These dimensions were based on the average diaphysis properties of the femurs and were kept constant for each specimen.

### Soft Tissue Shape and Positioning into a Sideways Fall Alignment

Soft tissue shapes were generated using the same reference soft tissue shapes from a database used by Fleps et al. [18] In the database, the soft tissue scans were already annotated with estimated knee and pelvis locations. Using this, the pelvis was placed inside the soft tissue with the experimentally measured pelvis tilt (5.50° to 18.78°). [15].

**Fig. 3:**
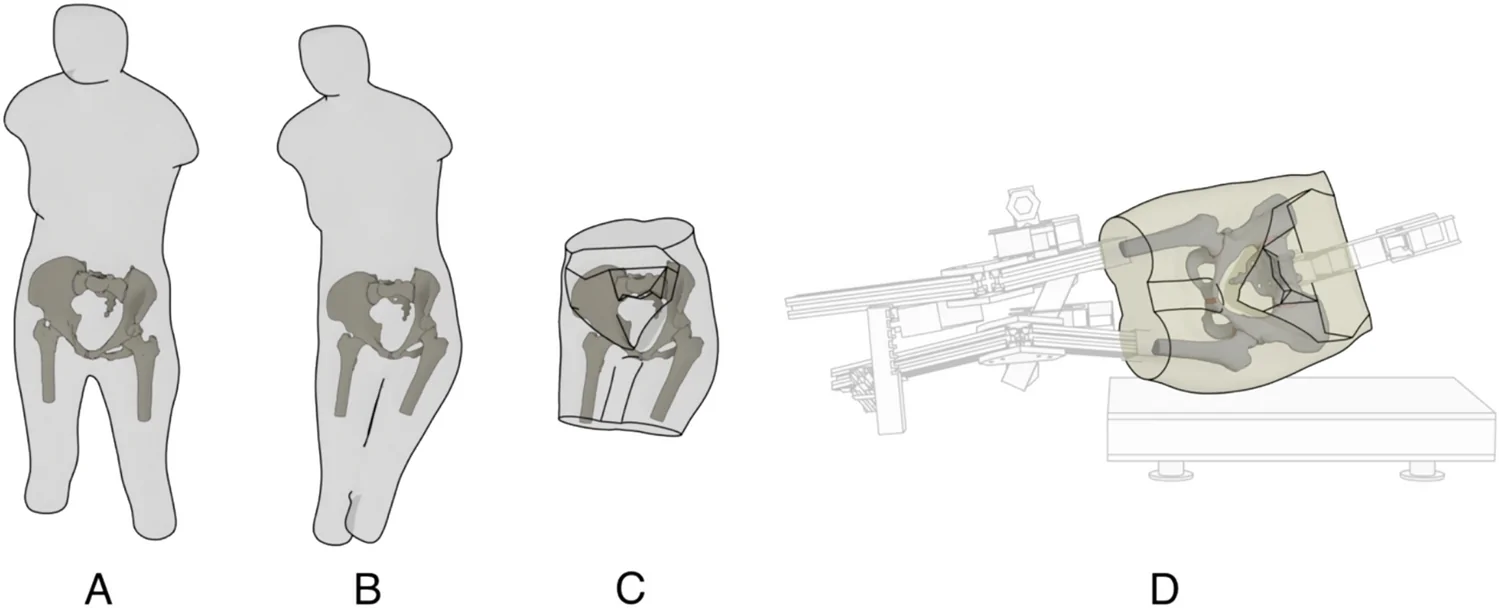
Soft tissue morphing from an upright position into the fall setup. **A** The bones are placed in the soft tissue. **B** The soft tissue is morphed into the fall alignment. **C** The soft tissue is truncated to the experimental equivalent region. **D** The soft tissue is placed into the fall setup

Each soft tissue was morphed into a fall configuration using an implicit FE simulation where the bones were placed inside the soft tissue and then simulated into position. First, the bones were placed within the upright positioned soft tissue (Fig. 3A). In this position, the soft tissue was expanded or contracted in the transverse plane by a transverse scaling factor to adjust the final inertia. The bones were then positioned into the fall alignment and the soft tissue was allowed to deform (Fig. 3B). In this way, the soft tissue realistically deformed into a fall configuration.

The soft tissue above the greater trochanter of the impacted side was morphed to the targeted soft tissue thickness as targeted by Fleps et al. and the entire soft tissue was truncated to the same size [18]. (Fig. 3C). Next, it was meshed with a target element size of 1mm. The soft tissue was subsequently tied to the sleeves, femurs, and pelvis so that no motion could occur between them (Fig. 3D).

Once in the fall alignment, the inertia was calculated. If the inertia was not within 0.01% of the target inertia, the entire soft tissue shape and positioning process was repeated with a new soft tissue transverse scaling factor. The target inertia was based upon the specimens’ weight. [18].

### Baseline Simulations

Each of the 11 specimens were run with no prescribed scan coverage errors and we refer to these as the “baseline” simulations. That is, each simulation had the full femur length, no shaft axes errors, and the pelvis from the source specimen. These simulations were used to evaluate how well the automated pipeline could replicate the experimental results. For these simulations, the error impact force (*E*_*BL*_) was compared directly against the experimental results reported by Fleps et al. [15].

## Limited CT Scan Coverage Simulations

### Input Parameters

In order to investigate the effects of limited CT scan coverage, we introduced various scan coverage input errors into models. The types of errors and differences between the models introduced were split into several categories:

1. Length of scan coverage, *L*_*S*_. This defines how much the femur needs to be extended to connect with the metal sleeve.
2. Shaft estimation error, both direction (θ) and magnitude (ϕ)
3. Choice of template pelvis.

### Femur Length

**Fig. 4:**
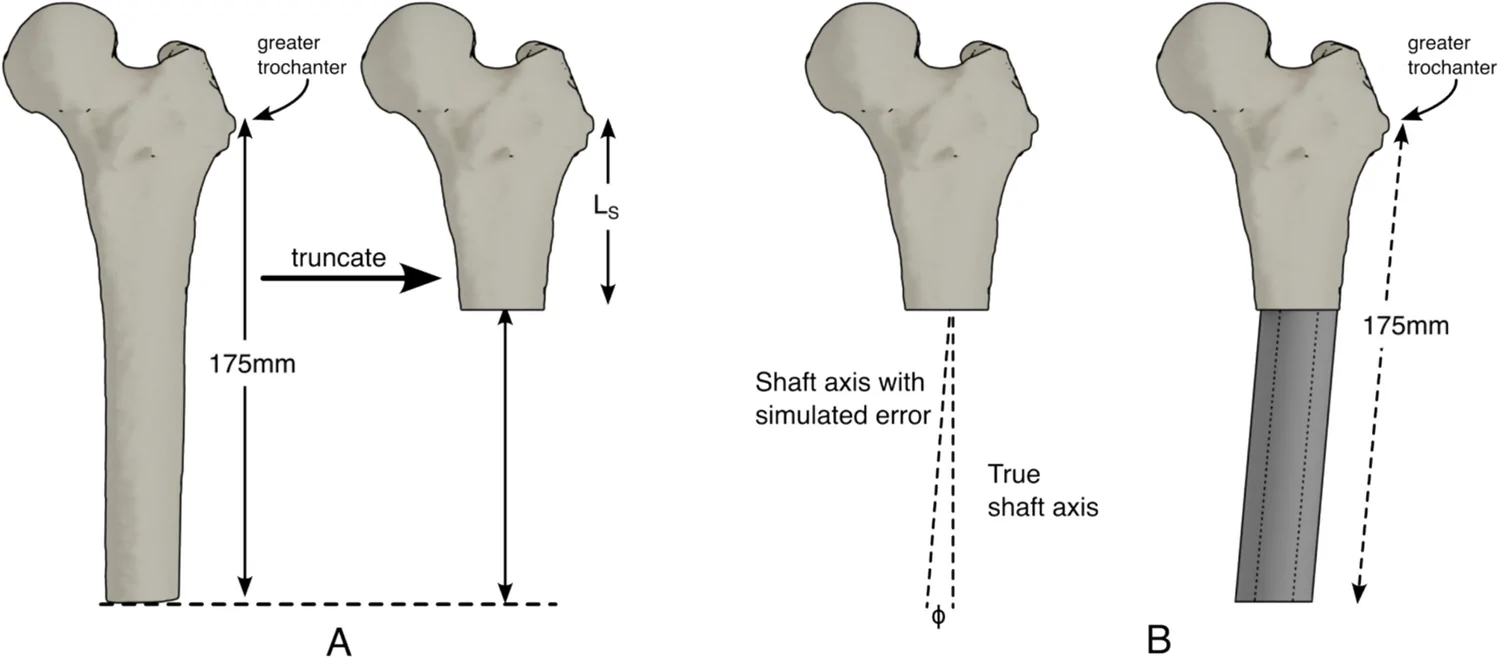
**A** The scan coverage was simulated by truncating the femur along the shaft, which was determined by the experimental placement of the sleeve. The truncation determines the Scan Length, L_S_, past the lateral aspect of the greater trochanter. **B** The shaft directional error is introduced by setting a directional angle (θ) and magnitude (Φ). Here, (θ) is 90° and Φ is 5°

We simulated limited CT scan coverage by truncating the original femurs in the experimental shaft direction. These were then subsequently re-meshed and material mapped according to the protocols of the experimental setup (Fig. 4).

### Shaft Axes Direction Error

**Fig. 5:**
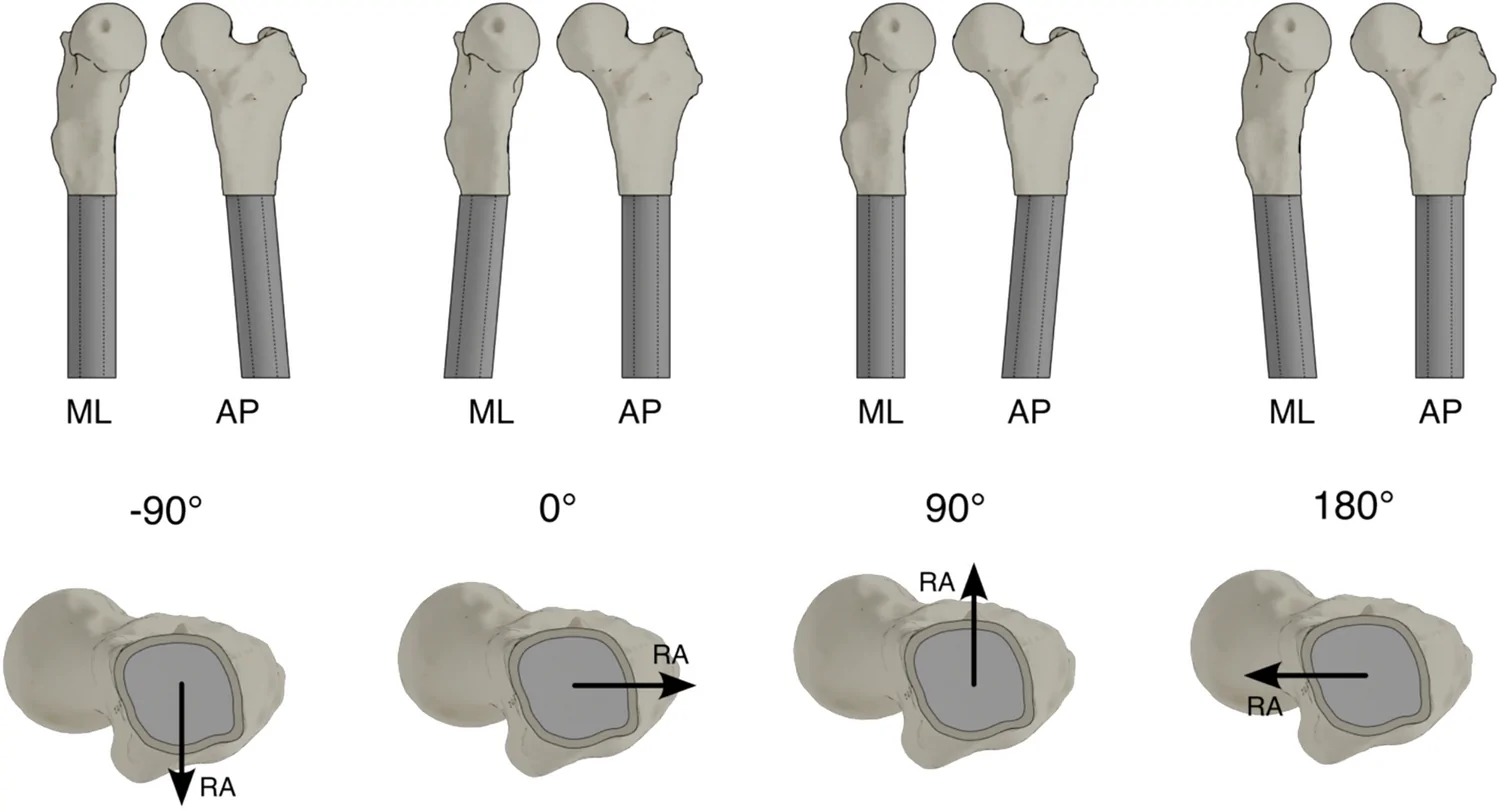
Examples of resulting shafts after shaft error introduction. Here, the rotation magnitude (Φ) is 5° to show the maximum directional error in the shaft axis. The rotation axis (RA) of error (θ) was varied in the experiment from − 180° to 180°

Error in the shaft axis direction was introduced by calculating a direction of error (θ) and a magnitude (ϕ). This error was added to the experimentally measured shaft axis of the impacted femur only (Fig. 5). This affected the relative angle of the pelvis to the femur along with the angle between the femur and the femur extension. This changes the angle of the femur relative to the impact surface.

### Pelvis Template Choice

To simulate not having a full pelvis in the CT scan, the pelvis of the simulated specimen was not used and instead we used a template pelvis, which was a pelvis from another specimen. These template pelvises were mismatched to the femurs in the scan and were subsequently morphed using the previously mentioned procedure.

Male and female pelvises were not mixed and matched, i.e., female template pelvises were morphed to female specimens and male pelvises were morphed to male specimens. In the male simulations, each of the 5 specimens were morphed to one another. In this way, there were 36 different pelvis–specimen combinations for females and 25 different pelvis–specimen combinations for males.

### Output Measures

**Fig. 6:**
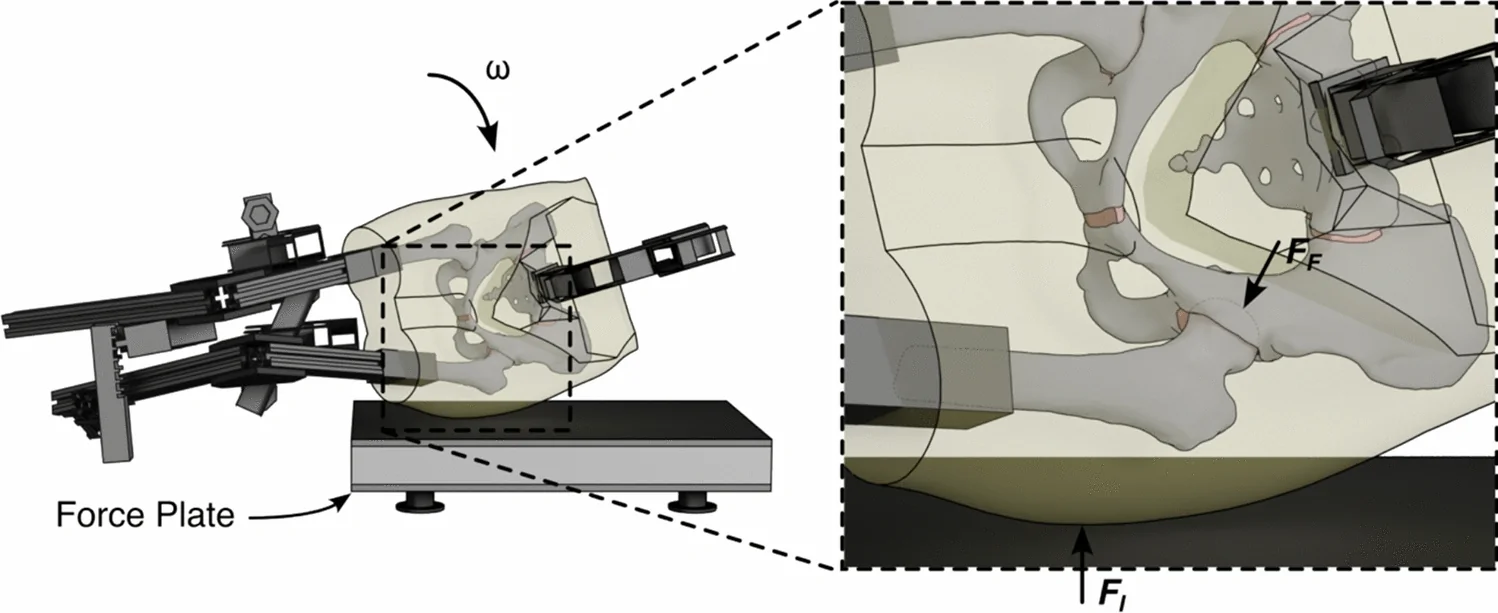
Biofidelic simulator FE model at impact with initial rotational velocity ω. The Impact Force (F_I_) is the force between the soft tissue and the force plate. The Femur Force (F_F_) is the force between the pelvis and the left femur

The output measure error was calculated as the difference between the output measure from the baseline FE models, i.e., those without simulated scan coverage, and the FE models with simulated scan coverage. Five output measures were considered: Impact Force Error (*E*_*I*_), Femur Force Error (*E*_*F*_), Log Fragility Ratio Error of the Impact Force (*E*_*FRI*_), and Log Fragility Ratio of the Femur Force (*E*_*FRF*_) (Fig. 6).

The impact force was the force measured by the force plate, and the femur force was the force between the pelvis and the femur on the impacted side. The impact force was previously measured experimentally [17, 18], while the femur force was not.

The fragility ratio is the ratio of the peak force in a simulation using elastic bone material properties to the same peak force in an equivalent simulation but with nonlinear bone material properties. It characterizes how far the simulation has gone into the nonlinear and strain-softening (i.e., damage-like) regions. In this study, we used the log transform as it improves interpretability and makes the measure behave more linearly. Thus, the log fragility ratio is defined as follows:

$$\log \left[\frac{{\mathrm{max}}(F_{elastic})}{{\mathrm{max}}(F_{nonlinear})}\right]$$

Where *F* is the impact force or the femur force. Higher values indicate higher risk of fracture. For reference, a fragility ratio of 1.3 was identified as a discriminating threshold in our previous work and this corresponds to a log fragility ratio of 0.26. [5].

### Parameter Study Design

A Latin Hypercube Design was used to investigate all the input error parameters. Briefly, a Latin hypercube experimental design is a space-filling experimental design that evenly spreads the input parameters to fill as much of the space as possible [23]. This decreases the number of simulations that need to be run, and, while not strictly enforced, each input parameter is approximately orthogonal to one another.

For the male simulations, each of the 25 pelvis–specimen combinations had 20 evenly distributed combinations of shaft direction error and scan lengths. In total, there were 500 male simulations. Similarly, in the female simulations, each of the 36 pelvis–specimen combinations had 20 evenly distributed combinations of shaft direction error and scan lengths. In total, there were 720 female simulations.

Altogether, 1220 models were built and, for the calculation of the fragility ratios, each was simulated twice for the parameter study—once with nonlinear bone material properties and once with elastic bone material properties. This resulted in 2440 simulations in total. Each of the 1220 models was treated as an observation during analyses.

### Statistical Analyses

All statistical analyses and experimental design were conducted using R (4.2.1). Inference was performed using multiple linear regression with each input (Table 2) being a predictor and each output measure being the dependent variable. Variance explained for each predictor was calculated by including only the relevant term into the simple linear regression model and recording the explained variance (R [24]). Additionally, a fully saturated model was created utilizing all predictors.

**Table 2:** Ranges of input parameters investigated

Parameter	Range
Femur shaft error direction, θ	−180° to 180°
Femur shaft error magnitude, ϕ	0° to 5°
Length of scan coverage, *L*_*S*_	25 mm to 170 mm distal to the lateral aspect of the greater trochanter
Pelvis Source	11 specimens

As the inputs are, by design, orthogonal to one another, the sum of each individual predictors’ explained variance totals the variance explained of the fully saturated model—although only approximately as the pairwise correlations were not strictly controlled to be zero.

## Results

All baseline models (N = 11) were simulated without issue. For the parameter study, 49 (2.0%) simulations of the 2440 simulations did not finish for various reasons with the most common reason being a failure during the automated simulation build process. As the fragility ratio combines the elastic material model simulation and the nonlinear bone material model simulation, the observation was dropped if either of them was missing leaving a total of 1181 (96.8%) valid observations of the original 1220.

### Baseline Simulations

In the baseline simulations, the peak impact forces ranged from 3.85 to 7.64 kN with male simulations producing higher forces than female simulations (Table 3). The baseline simulations had peak impact forces that were comparable to those of the experiments [15, 18] with mean impact force difference (*E*_*BL*_) of − 0.007 kN (σ= 0.409 kN, RMSE = 0.388 kN).

**Table 3:** Tabulated results of the 11 baseline simulations. Of these output measures, only the impact force (F_I_) was able to be measured experimentally

Specimen	Impact force, *F*_*I*_ [kN]	Impact force error, *E*_*BL*_ [kN]	Femur force, *F*_*F*_ [kN]	Log fragility ratio impact Force, *FR*_*I*_ [-]	Log fragility ratio femur force, *FR*_*F*_ [-]
H1389	3.850	−0.903 (− 30.6%)	2.394	0.615	0.773
H1390	5.566	0.565 (9.2%)	3.687	0.306	0.358
H1391	5.831	−0.190 (− 3.4%)	3.416	0.107	0.216
H1392	5.005	−0.098 (− 2.0%)	2.648	0.312	0.472
H1394	4.831	0.127 (2.6%)	2.214	0.236	0.471
H1395	4.937	−0.027 (− 0.6%)	1.902	0.036	0.078
H1397	5.277	−0.035 (− 0.7%)	3.766	0.422	0.463
H1399	5.056	−0.013 (− 0.3%)	3.436	0.442	0.508
H1401	5.140	− ^a^	3.342	0.469	0.602
H1402	7.645	−0.044 (− 0.6%)	5.299	0.134	0.147
H1406	6.582	0.550 (7.7%)	4.105	0.363	0.495

E_BL_ represents the error of the baseline model with respect to the experiment and the percentages are reported with respect to the experimental measurements

^a^ Not reported in Fleps et al due to instrument failure [15]

### Limited CT Scan Coverage Parameter Study

**Fig. 7:**
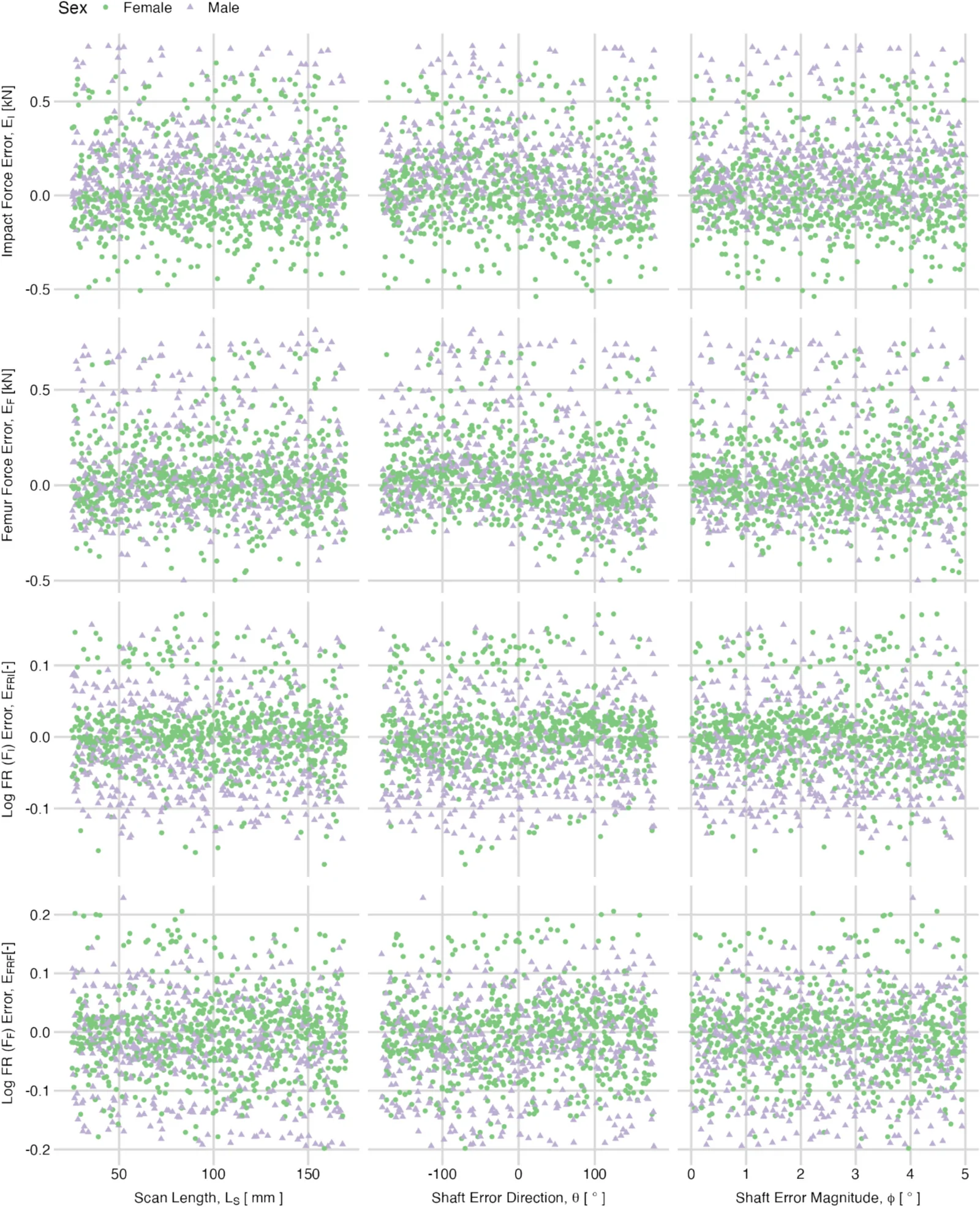
Impact Force Error (E_I_), Femur Force Error (E_F_), Log Fragility Ratio from Impact Force (E_FRI_), and Log Fragility Ratio from Femur Force (E_FRF_), for males and females across different scan lengths (L_S_), shaft error directions (θ), and different shaft error magnitudes (Φ)

Overall, the female observations with limited CT scan coverage showed lower average errors than the male simulations, but the scatter of the errors was similar (Table 4). *E*_*I*_, *E*_*F*_*, E*_*FRI*_*, and E*_*FRF*_ showed very small correlations with *L*_*S*_, θ, or ϕ (Fig. 7).

**Table 4:** Mean and standard deviation (σ) for error of impact force, femur force, fragility ratio using impact force (E_FRI_), and fragility ratio using femur force (E_FRF_) across all observations of the parameter study

	Unit	Male	Female	Total
Impact force error, *E*_*I*_	kN	0.145 (0.217)	0.034 (0.231)	0.081 (0.232)
Femur force error, *E*_*F*_	kN	0.093 (0.267)	0.042 (0.195)	0.063 (0.228)
Log fragility ratio (*F*_*I*_) error, *E*_*FRI*_	–	−0.018 (0.064)	0.005 (0.051)	−0.004 (0.058)
Log fragility ratio (*F*_*F*_) error, *E*_*FRF*_	–	−0.027 (0.080)	−0.002 (0.068)	−0.012 (0.074)

Across all simulations, the error of the femur force (*E*_*F*_) was lower than the error of the impact force (*E*_*I*_), and the fragility ratio calculated from the impact force (*E*_*FRI*_) was lower than the fragility ratio calculated from the femur force (*E*_*FRF*_). Summary results are tabulated in Table 4.

**Fig. 8:**
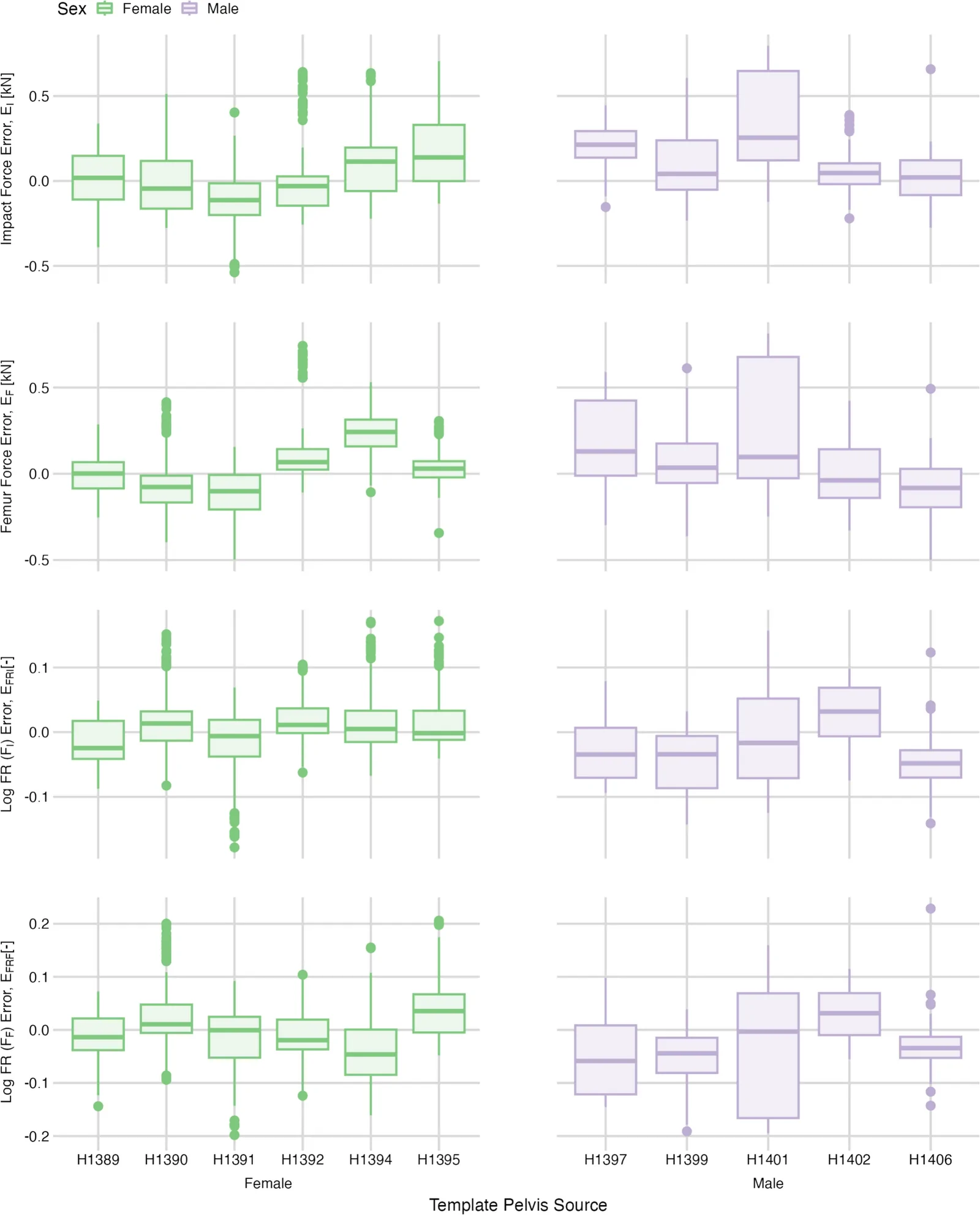
Boxplots of the outcome measure errors across different source pelvises for female simulations (left) and male simulations (right). The central line represents the median, the edges of the box indicate the first and third quartiles, and the whiskers extend to 1.5 times the interquartile range. Outliers are shown as individual points beyond the whiskers

Each outcome measure varied depending on the source pelvis used. For *E*_*I*_, by source pelvis (Figure 8), standard deviations ranged from 0.189 kN (H1389) to 0.229 kN (H1392) for females; 0.113 kN (H1402) to 0.279 kN (H1401) for males. For *E*_*F*_, by source pelvis, standard deviations ranged from 0.099 kN (H1395) to 0.228 kN (H1392) for females; 0.156 kN (H1406) to 0.360 kN (H1401) for males. For *E*_*FRI*_, by source pelvis, standard deviations ranged from 0.031 (H1389) to 0.057 (H1394) for females; 0.0433 (H1406) to 0.087 (H1401). For E_FRF_, by source pelvis, standard deviations ranged from 0.046 (H1406) to 0.120 (H1401). The summary metrics grouped by source pelvis is tabulated in Table 5.

**Table 5:** Mean and standard deviation (σ) of the outcome measures grouped by source pelvis used along with the number of complete observations. Note that due to the experimental design, each female pelvis had 120 models and each male pelvis had 100 models

Source pelvis	Impact force error, *E*_*FI*_	Femur force error, *E*_*FF*_	Log fragility ratio (*F*_*I*_) error, *E*_*FRI*_	Log fragility ratio (*F*_*F*_) error, *E*_*FRF*_	Complete observations (%)
	kN	kN	–	–	–
Female					
H1389	0.001 (0.189)	0.001 (0.122)	−0.019 (0.031)	−0.014 (0.044)	119 (99.1)
H1390	−0.001 (0.204)	−0.051 (0.173)	0.018 (0.055)	0.028 (0.072)	116 (96.7)
H1391	−0.122 (0.190)	−0.113 (0.144)	−0.021 (0.054)	−0.022 (0.064)	118 (98.3)
H1392	0.023 (0.229)	0.151 (0.228)	0.019 (0.037)	−0.014 (0.051)	119 (99.1)
H1394	0.131 (0.227)	0.237 (0.135)	0.018 (0.057)	−0.033 (0.072)	113 (94.2)
H1395	0.181 (0.215)	0.035 (0.099)	0.018 (0.049)	0.046 (0.063)	114 (95.0)
Male					
H1397	0.209 (0.127)	0.191 (0.240)	−0.026 (0.051)	−0.049 (0.077)	99 (99.0)
H1399	0.093 (0.177)	0.065 (0.180)	−0.045 (0.049)	−0.053 (0.052)	99 (99.0)
H1401	0.366 (0.279)	0.276 (0.360)	−0.001 (0.087)	−0.033 (0.120)	96 (96.0)
H1402	0.056 (0.114)	0.001 (0.179)	0.027 (0.044)	0.031 (0.047)	100 (100.0)
H1406	0.018 (0.141)	−0.082 (0.156)	−0.047 (0.043)	−0.030 (0.046)	88 (88.0)

Across all outcome measures, the pelvis–specimen interaction term explained the most variance. For *E*_*I*_, the pelvis–specimen interaction explained 87.6% of the total variance (Table 6); for *E*_*F*_, 88.0% (Table 7); for *E*_*FRI*_, 90.1% (Table 8); and for *E*_*FRF*_, 88.4% (Table 9).

**Table 6:** Impact Force Error, E_I_, variance explained by the predictors scan length (L_S_), shaft error direction (θ), shaft error magnitude (ϕ) and pelvis specimen interaction (P:S) in linear regression models

Regression Model	R [24]
$E_I \sim L_S$	0.0002
$E_I \sim \theta$	0.0050
$E_I \sim \phi$	0.0003
$E_I \sim P:S$	0.8763
$E_I \sim L_S+\theta +\phi +P:S$(saturated model)	0.8861

**Table 7:** Femur Force Error, E_F_, variance explained by the predictors scan length (L_S_), shaft error direction (θ), shaft error magnitude (ϕ) and pelvis specimen interaction (P:S) in linear regression models

Regression Model	R [24]
$E_F \sim L_S$	0.0006
$E_F \sim \theta$	0.0223
$E_F \sim \phi$	0.0000
$E_F\sim P:S$	0.8801
$E_F \sim L_S+\theta +\phi +P:S$(saturated model)	0.9054

**Table 8:** Fragility Ratio Error—Impact Force, E_FRI_, variance explained by the predictors scan length (L_S_), shaft error direction (θ), shaft error magnitude (ϕ) and pelvis specimen interaction (P:S) in linear regression models

Regression Model	R [24]
$E_{FRI} \sim L_S$	0.0002
$E_{FRI} \sim \theta$	0.0011
$E_{FRI} \sim \phi$	0.0000
$E_{FRI} \sim P:S$	0.9014
$E_{FRI} \sim L_S+\theta +\phi +P:S$(saturated model)	0.9110

**Table 9:** Fragility Ratio Error—Femur Force, E_FRF_, variance explained by the predictors scan length (L_S_), shaft error direction (θ), shaft error magnitude (ϕ) and pelvis specimen interaction (P:S) in linear regression models

Regression Model	R [24]
$E_{FRF} \sim L_S$	0.0007
$E_{FRF} \sim \theta$	0.0043
$E_{FRF} \sim \phi$	0.0003
$E_{FRF}\sim P:S$	0.8837
$E_{FRF} \sim L_S+\theta +\phi +P:S$(saturated model)	0.8987

## Discussion

In this study, we automated the construction of biofidelic sideways fall models, compared the baseline results to the experimental results, and investigated the role of scan coverage, shaft direction estimation, and using a template pelvis on key outcome measures that may play a role in understanding and predicting hip fractures. We found that we were able to replicate the experimental results, the scan coverage errors were acceptably small, and that the choice of template pelvis influenced the results the most.

The baseline simulations, which were generated using an automated pipeline, generally agreed with the experimental results. With the exception of H1389, which was the smallest specimen, the automated pipeline is a good and relatively unbiased estimator of the experimental results. The mean impact force error of the baseline models$({E}_{BL})$ was slightly lower than the mean error reported by Fleps et al. (− 0.165 kN; σ=0.506 kN). The models in this study were built to the same specifications; however, the simulations in Fleps et al. were manually built while the simulations built for this study were fully automated. As such, small differences exist in the models, with the largest differences seen in the shape of the soft tissue. The femur force matched well with the simulation results from Fleps et al., with a mean difference of 0.203 kN (σ = 0.217 kN). These results indicate that the base simulations, which here were built using an automated pipeline, are equivalent to those that were previously built for impact and femur forces.

Across all observations in the parameter study, the impact force error, *E*_*I*_, was relatively small. The average impact force error was 0.081 kN (SD 0.232 kN), which compared to the average impact force for all simulations of 5.429 kN, represents a mean error of 1.5% and standard error of 4.3%. This was not unexpected as the impact force is predominantly a function of the initial momentum and the effective stiffness of the system. The momentum of the system was, by construction of the system, kept constant through a target inertia. Furthermore, the main loading path during the impact was through the soft tissue, greater trochanter, and finally into the pelvis. No matter the scan length, or the simplistic nature of the femur extension, the soft tissue thickness was kept constant and the greater trochanter was always included. Thus, the effective stiffness of the system remains similar throughout all the simulations.

Compared to the impact force errors, the femur force errors were slightly larger. The femur force, which is the contact force between the pelvis and the impact side femoral head, is more dependent on the load coming from the pelvis. In this case, the contralateral femur and pelvis is pushed onto the femoral head. As such, the pelvis likely plays a larger role—exacerbating the differences in pelvis shape from a morphed pelvis.

Errors in the log fragility ratios calculated using the impact force and femur force were also relatively small. Across all simulations, the standard deviations were only 0.058 (*E*_*FRI*_) and 0.074 (*E*_*FRF*_), which is almost an order of magnitude smaller compared to the average value in the baseline simulations (*FR*_*I*_ 0.313; *FR*_*F*_ 0.417). The fragility ratio is a surrogate measure of fracture status, and these small values indicate that the amount of nonlinear material behavior is not affected substantially by the introduced scan coverage errors.

The saturated linear regression models accounted for most of the variance observed in the output. The unexplained variance is due to nonlinear and interaction terms between the predictors which were omitted to simplify the interpretation.

Compared to scan length and shaft errors, the choice source pelvis choice played a much larger role. The pelvis choice also interacted with the source specimen as well, meaning that the combination of specimen/pelvis rather than pelvis choice alone, usually explained almost all the variance (87.6% for *E*_*I*_, 88.0% for *E*_*F*_, 90.1% for *E*_*FRI*_ and 88.4% for *E*_*FRF*_). This indicates that the morphing procedure we used introduces much more outcome measure error into the simulations than the femoral scan coverage or errors of shaft direction.

Furthermore, some pelvises tended to have lower errors across all specimens, while other had lower error for some specimens but not for others. For female subjects, the pelvis from H1389 generally performed the best with the lowest mean error, and a generally low scatter. More importantly, the pelvis from H1389 was very robust during the building process and was able to be simulated in almost every condition. For male subjects, the pelvis from H1406 performed the best and had the lowest mean errors and scatter compared to the other pelvises. However, H1406 had the most issues during the model building process and only 88% of the targeted conditions were successfully simulated. H1402 had slightly higher scatter, however, 100% of the targeted conditions were successfully simulated indicating high computational robustness of this pelvis. These results indicate that, if a generic template pelvis is needed, the pelvis from H1389 should be used for female subjects and the pelvis from H1402 should be used for male subjects.

We found that shaft directional errors (θ, ϕ) introduced relatively small errors in all measured outputs. With respect to the magnitude of orientation errors introduced, Zhang et al. investigated errors from partial femur coverage and found that mean orientation errors averaged 5.44° ± 2.81° (abduction/adduction) and 5.34° ± 2.82° (flexion/extension) [25], which is only slightly larger than the shaft errors investigated in this study. The effect of shaft error may be minimized because the inertia and positioning of the rest of the specimen is more driven by general inertia and system stiffness rather than femur orientation. In practice, the scan length would likely be correlated with shaft directional errors as the ability to estimate the shaft direction becomes more difficult with less scan available.

Interestingly, not all the variance output was explained by the input parameters. Only 88.61%–91.10% of the output parameter variance was explained by the first-order models. The unexplained variance could be due to higher order and interaction terms, but, more likely, is the inherent uncertainty associated with complex nonlinear dynamic modeling. Despite the fact that these simulations are deterministic problems, and that the results do not change under mesh refinement (i.e., mesh convergence) [24], small input perturbation still changes the discretization which affect the simulation outputs. In the case of the femur force, this corresponds to a relative uncertainty of ± 1.78%.

This study is mainly limited by the relatively small sample size of the source specimens. Although good agreement was found between the simulations and experiments, more specimens should be tested to verify these results. Furthermore, while we felt these sources of error would be the most dominant and common, there are other sources of error that we did not consider including CT scanner differences, phantom calibration differences, and segmentation variability. CT scanners have been shown to affect the density values independent from calibration differences [26] and could introduce an additional unaccounted for error in future in-silico trials. Furthermore, phantomless calibrations, while showing relatively good inter-operator reliability [27], can affect the subsequent bone material properties. Thus, calibration differences could have an influence on future in-silico trial results and should be investigated.

Because errors are explained most by the pelvis–specimen interaction, future work should focus on improving the template pelvis shape used or including more measures of pelvis error as predictors. For example, rather than morphing another specimen’s pelvis to match the critical measurements of another specimen, statistical shape modeling could be used to reconstruct that particular specimen from limited scan coverage or anthropometric variables [28]. Furthermore, it remains to be seen if the FE modeling is predictive of fracture in a clinical setting. While earlier dynamic modeling attempts have shown promise [5], it is unclear if the automated methodology here is predictive as well and how the errors presented here would propagate with respect to predictive fracture classification accuracies.

We built an automated workflow to construct biofidelic sideways fall models and found that it matched well with experimental results. Furthermore, the effects of limited CT scan coverage played relatively small roles with respect to force measurement outcomes. The source template pelvis played the largest role, while the femur length played a very small role. Future translational studies should take care to investigate whether their chosen pelvis adequately represents the specimen to be investigated. However, if an appropriate pelvis is chosen, then this biofidelic setup provides a foundation to explore larger clinical cohorts in silico to determine if this sideways fall setup is predictive of real-world hip fracture.

## Supplementary Information

Below is the link to the electronic supplementary material.

Supplementary file1 (CSV 197 KB)

## Data Availability

The datasets generated and analyzed during the current study are available as supplemental material on this publication.
